# Normal values of the maximal respiratory pressures in healthy people older than 20 years old in the City of Manizales - Colombia

**Published:** 2012-06-30

**Authors:** Lida Maritza Gil Obando, Alexandra López López, Carmen Liliana Ávila

**Affiliations:** 1 Member of the Research Group Body and Movement. Department of Human Movement Sciences Universidad Autónoma de Manizales. E-mail: lidagil@autonoma.edu.co; 2 Member of the Research Group Body and Movement. Department of Human Movement Sciences Universidad Autónoma de Manizales. E-mail: alexlo@autonoma.edu.co; 3 Member of the Research Group Body and Movement. Department of Human Movement Sciences Universidad Autónoma de Manizales. E-mail: liliavila@autonoma.edu.co

**Keywords:** Respiratory muscles, muscle strength, maximal respiratory, pressures predictive equations, reference values

## Abstract

**Introduction:**

The Maximal Inspiratory Pressure (MIP) and Maximal Expiratory Pressure (MEP) are global measures of the maximal strength of the respiratory muscles.

**Objectives:**

To determine the values of MIP and MEP in healthy subjects aged 20 years old from the urban area of Manizales, Colombia and to correlate them with sociodemographic and anthropometric variables.

**Methods:**

This is an observational descriptive study. The population of the study was 203,965 healthy people from Manizales, a Colombian city located at 2,150 meters above sea level. The sample size was 308 subjects, selected using simple random sampling. The maximal respiratory pressures were determined in the sample chosen and were then considered according to the variables of age, gender, size, weight, Body Mass Index (BMI), and BMI classification. Finally a predictive model was created.

**Results:**

The average MIP value among the subjects of the study was 75±27 cmH20 and the MEP value was 96.4±36 cmH20. Both averages were higher in men than in women. Predictive equations were established for the normal values of MIP and MEP in healthy subjects; the best model for MIP was the resultant one among age, gender and BMI classification and for the MEP among gender, weight and height.

**Conclusion:**

Maximal respiratory pressure values were lower among the population of Manizales than those found in international studies. Gender and anthropometric characteristics (weight, height and BMI classification) are the explanatory variables that better support the average values of MIP and MEP in the predictive models proposed.

## Abstract (es)

**Introducción:**

La Presión Inspiratoria Máxima (PIM) y Presión Espiratoria Máxima (PEM) son medidas globales de la fuerza máxima de los músculos respiratorios.

**Objectivos:**

Determinar los valores de PIM y PEM en sujetos sanos mayores de 20 años de la zona urbana de Manizales-Colombia y correlacionarlos con variables sociodemográficas y antropométricas.

**Métodos:**

Estudio observacional descriptivo. La población referente fueron 203,965 personas sanas de Manizales, ciudad colombiana ubicada a 2,150 metros sobre el nivel del mar. El tamaño muestral fue de 308 sujetos, se llevó a cabo un muestreo aleatorio simple. Se determinaron las presiones respiratorias máximas en la muestra seleccionada y se relacionaron con las variables edad, género, talla, peso, Índice de Masa Corporal (IMC), y clasificación de IMC, finalmente se construyó un modelo predictivo.

**Resultados:**

El valor promedio de PIM en los encuestados fue de 75±27 cmH20 y el de PEM de 96.4±36 cmH20, ambos promedios mayores en los hombres que en las mujeres. Se establecieron las ecuaciones de predicción para los valores normales de PIM y PEM en sujetos sanos; el mejor modelo para la PIM fue el resultante entre edad, género y clasificación de IMC y para la PEM entre género, peso y talla.

**Conclusión:**

Se evidencian valores de presiones respiratorias máximas inferiores en población Manizaleña que los encontrados en estudios realizados a nivel internacional. El género y las características antropométricas (peso, talla y clasificación de IMC) son las variables explicativas que mejor soportaron los valores promedio de PIM y de PEM en los modelos predictivos propuestos.

## Introduction

Maximal inspiratory pressure (MIP) and maximal expiratory pressure (MEP) are global measures of maximal strength of respiratory muscles and they are respectively the greater pressure which may be generated during maximal inspiration and expiration against an occluded airway^3^. The MIP is a measure of inspiratory muscle strength produced by a sub-atmospheric pressure and the MEP is a supra-atmospheric pressure which can be developed in an effort of the abdominal and intercostal muscles^4^.

In 1969, Black and Hyatt^5^ introduce a simple way to measure maximal respiratory pressures with a hand-held mouth pressure meter in cmH2O. This is a way to quantitatively measure the function and respiratory muscle strength; since then respiratory muscle strength has been measured by the determination of maximal respiratory pressures, specifically by the generation of static maximum pressures in the mouth against an occluded airway. This is indicative of the strength of inspiratory and expiratory muscle groups^1^.

This research project was based on the physiological concept of maximal strength of respiratory muscles and their determinants such as age, gender, anthropometric characteristics, barometric pressure or restrictive^6^ or obstructive pathology. Measuring MIP and MEP is a simple, rapid, non invasive, validated, and widely used in evaluating respiratory muscle function.

Given the importance of measuring maximal respiratory pressures, especially in cardiopulmonary and neuromuscular areas, several studies have attempted to establish predictive values of MIP and MEP. Black and Hyatt^5^ described a method of the assessment of respiratory muscle strength in 120 healthy subjects of both sexes aged between 20 and 86. This determined the values of maximal respiratory pressures and reference equations for healthy population. Using variables such as age, sex and, after that first study, several authors evaluated the MIP and MEP in healthy people of different races^7^, ages^8^ ^-^ ^10^ and published the results of the reference values of the predictive equations for the calculation of maximal respiratory pressures.

Camelo Jr. *et al* ^11^ were the first to describe the values of MIP and MEP in a Brazilian population sample of 60 healthy subjects of both sexes aged between 20 and 49.5 years. Johan *et al*. ^7^, conducted PEM and PIM studies on Asian people to define normal values for Chinese, Malay and Indian adults. They concluded that differences in lung function among these ethnic groups are found in the respiratory muscle strength, lung elastic recoil, alveolar and airway growth and compliance of the thoracic cavity and the dimensions of the chest wall. Neder *et al*. ^3^, evaluated 100 healthy subjects of both sexes aged between 20 and 80 years. These authors proposed a regression analysis, pioneered the development of predictive equations for MIP and MEP dependent on sex and age based on a Brazilian population sample. Parreira *et al*. ^2^, concluded that the equations predicted by Neder *et al*., were not able to predict values of MIP and MEP for specific populations. For that reason, every specific application must be carefully made in the clinical context. As a consequence, the American Thoracic Society state that the reference values of this important measure as well as of other biological variables should ideally be derived from a random selection, geographically related to the population to ensure greater accuracy and predictive power. If these cannot be met, the test results can cause interpretation errors^12^

In this sense, MIP and MEP values as found in research in the international context may be inappropriate for using them in the Colombian population, leading to little objective diagnosis of lung function. This implies taking into account morphophysiological differences such as race, gender, weight, height, BMI, which differ from one population to another and therefore can modify the results of these measures^13^

The objective of this study was to obtain MIP and MEP values in healthy subjects older than 20 years in the urban area of Manizales, Colombia, and to correlate them with anthropometric and sociodemographic variables.

Predictive equations for both MIP and MEP in healthy adult population of Manizales were established. It is important to have predictive formulas of maximal respiratory pressures validated in the Colombian context, because of the different anthropometric, environmental and cultural characteristics that can be used in the clinical practice and in the field of research in Colombia.

## Methods

This was a descriptive observational study. The population consisted of 203,965 subjects from the urban area of Manizales (Colombia), a city located at 2,150 meters above sea level. The initial sample size was 272 people. After a first analysis, variability of intergroup age was detected and the sample was adjusted in an additional margin of 13% in order to balance the subgroups according to gender (males 50% and females 50%) and age ranges (between 20 and 39 years: 50%; 40 years old and over: 50%). The final sample size was 308 subjects. A simple random sample was used with a confidence level of 95% and a margin of error of 2.7 ± 23.6 cmH2O for MIP, which was a quantitative variable that ensured greater sample size. The reference guide for the sample adjustment had been previously published by Rodríguez *et al*.^14^, that is, similar variables and samples were followed by this study.

The inclusion criteria were: people considered healthy based on anamnesis analysis and a general physical examination, sedentary people, both genders, all races, all socioeconomic levels, aged 20 years or older, and living in Manizales in the past two months or more. The exclusion criteria were: active smoker or former smoker of less than two years, previous restrictive or obstructive lung disease, body mass index lower than 18.5 or greater than 35 Kg/cm^2^, any acute disease at the moment of the test, structural deviations of the spine and thoracic cavity abnormalities, digestive hernia, recent postoperative thoracic and abdominal surgery, dyspnea from any cause, and difficulty understanding commands.

### Techniques and Procedures:

The calibration of the measuring equipment for the study variables was carried out. For the weight variable, an electronic scale (TANITA brand) calibrated with precision of 0.1kg every 7 days was used. Weight was recorded in light clothing and without shoes. For the height variable, the measuring rod was located in the laboratory where measurements were made. This variable was recorded without shoes in the inspiration phase. BMI was calculated using the formula BMI=Weight (kg) / Height^2 ^(cms). The numerical value was recorded and classified according to WHO standards. For the measurement of maximal respiratory pressures, a pressure gauge (MICROMEDICAL RPM brand, Micro Medical Limited, PO Box 6, Rochester, Kent ME1 2AZ UK), with a previous vacuum calibration cmH2O every 7 days, with a range of approximately 300 cmH2O of expiratory pressure and inspiratory pressure was used. Additionally, to prevent leakages and increased pressure within the oral cavity, a Speedo nose clip with a standard pressure for adults and a mouthpiece made of silicone attached to the plastic tube of the manometer were used (part of Micro Medical Limited, gauge manufacturer).

Researchers were trained with the purpose of undertaking the test to measure maximal respiratory pressures. The test for measuring MIP and MEP requires understanding, collaboration and coordination of participants. For this reason, all subjects were instructed about the appropriate way to do it. Besides, a demonstration of the procedure was also carried out. The presence of leakages was prevented by checking that lips were firmly sealed around the mouthpiece and by using a nose clip to control the disturbance of the inspiratory and expiratory measurements by assisting the facial muscles. For the measurement of maximal respiratory pressures, important variables such as the endurance of the inspiratory and expiratory muscles and their variation according to body position should be considered. Thus, the participant was placed in a sitting position, with the hip at an angle of 90 degrees and feet flat on the floor.

The developed procedure began with a pilot test in which 30 people were included. The data collection instruments were adjusted. Three reviewers collected the data. The first one was responsible for the reading and signing of the informed consent (approved by the Ethics Committee of the Autonoma University of Manizales) and for the registration of the sociodemographic variables, the second one assessed the anthropometric variables, and the third one was in charge of the procedure and the recording of respiratory pressures. For the MIP determination, the participant was asked to introduce and adjust the mouthpiece and nose clip based on the residual volume and also to perform a maximal inspiration during 3 or 4 seconds. For the MEP determination from the total lung capacity, the subject was asked to introduce the mouthpiece and nose clip and perform a maximal exhalation for 3 or 4 seconds. Three MIP maneuvers and three MEP maneuvers were performed in a sitting position, recording the highest value in each of the three cases.

From the ethical point of view, this study was considered itself as a ''minimal risk research,'' according to Act 11 of resolution 008430 of 1993 of the Health Ministry of Colombia because of the noninvasive clinical tests that did not risk the physical and moral integrity of the participants. Additionally, this research project met the principles set forth in the Declaration of Helsinki issued in 1993 by the World Medical Association. Its interest is scientific, and at all times the integrity of the participants was protected. All cautions to respect their privacy and to minimize the impact of the study on their physical and mental integrity were taken.

### Statistical analysis:

For data processing the statistical package SPSS® (Statistical Package for Social Science) version 17.0 for windows 2008 was used. For qualitative variables proportions were calculated and for quantitative variables mean, range and standard deviation were calculated. Confidence intervals were determined at 95%. The bivariate analysis assessed the relationship of independence and homogeneity of the anthropometric variables with the values of maximal respiratory pressures. They used correlation coefficients according to the measuring level of the variable and to their normal or abnormal behavior (Kolmogorov -Smirnov). The statistical differences were determined with a significance level of 95% (*p* ≤0.05). Additionally, homogeneity tests were carried out by using student's t-tests and Mann-Whitney U tests according to normal or abnormal behavior of the variables. For nominal variables with more than two groups, Fisher's F tests (ANOVA) and Kruskal-Wallis H were used. A multiple linear regression model from the model evaluation tests was constructed.

## Results

308 subjects participated in the study (no subjects dropping out during the observation phase), with an average age of 41 ± 13.7 years, and an average height and weight of 65 ± 11.6 kg and 164 ± 8.5 cm respectively, most participants had a normal BMI. The gender variable showed 49.7% and 50.3% for males and females, respectively and BMI was classified as normal: 59.4%, overweight: 35.4% and mild obese: 5.2%

**Table 1:**
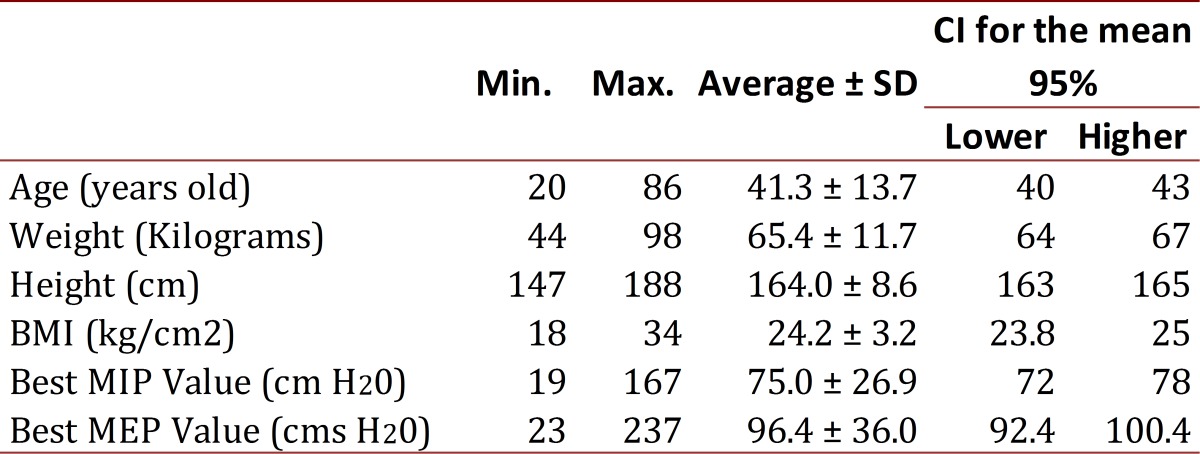
Descriptive analysis for quantitative variables. Manizales Colombia 2011 (n=308)

Tests were applied in order to verify compliance with assumptions (normality, homoscedasticity) for quantitative variables (MIP and MEP). The average MIP value among respondents was 75 ± 27 cmH20. According to the Mann Whitney U-test statistically significant differences were found (*p*= 0.000) between average MIP for males and females. Differentiating the variable subcategories, it was found that MIP average values in females (63.1 ± 20cm H20) were lower than in males (86.8 ± 28 cmH20). The MEP average value among respondents was 96.4 ± 36 cmH20, it was also lower in females (78 ± 24 cmH20) than in males (115 ± 37 cmH20). As with the MIP, MEP averages between males and females were significantly different (T= -10,394, *p*= 0.000) with a confidence interval of 95%.

Regarding MIP values and age, it was observed an inverse and significant correlation (r=- 0.161, *p* =0.005), which suggests that as age increases MIP values decrease. In reference to the relationship between age and the MEP, it is inverse (r= -0.096) and not statistically significant (*p*= 0.093). It was found that those with an age range of 20 to 39 years in both males and females, the MIP and MEP values were higher compared to those over 40 years.

The relationship between the MIP values and the weight is a regular positive relationship (r= 0.397) although statistically significant (*p*= 0.000). A similar situation happens with the relationship between the MEP values and the weight (r= 0.464, *p*= 0.000). For values of respiratory pressures and height, correlation is direct and significant for both, MIP (r= 0.436, *p*= 0.000) and MEP (r= 0.518, *p*= 0.000) values. BMI relationship to both, MIP and MEP values is direct and statistically significant (r= 0.188, *p*= 0.001 and r= 0.209, *p*= 0.000 respectively).

Kruskal-Wallis test was applied to establish whether there were differences between MIP values and BMI classification and it was found significant statistically differences in mean (*p*= 0.001). According to the BMI classification, it was found that MIP value was higher in mildly obese subjects (217.2 cmH20) than in overweight ones (167.4 cmH20) and those with normal weight (141.4 cmH20).

Considering MEP values and BMI classification, it was found that MIP and MEP values depend on anthropometric characteristics, biotype, nutritional status and physical fitness among populations.

**Table 2:**
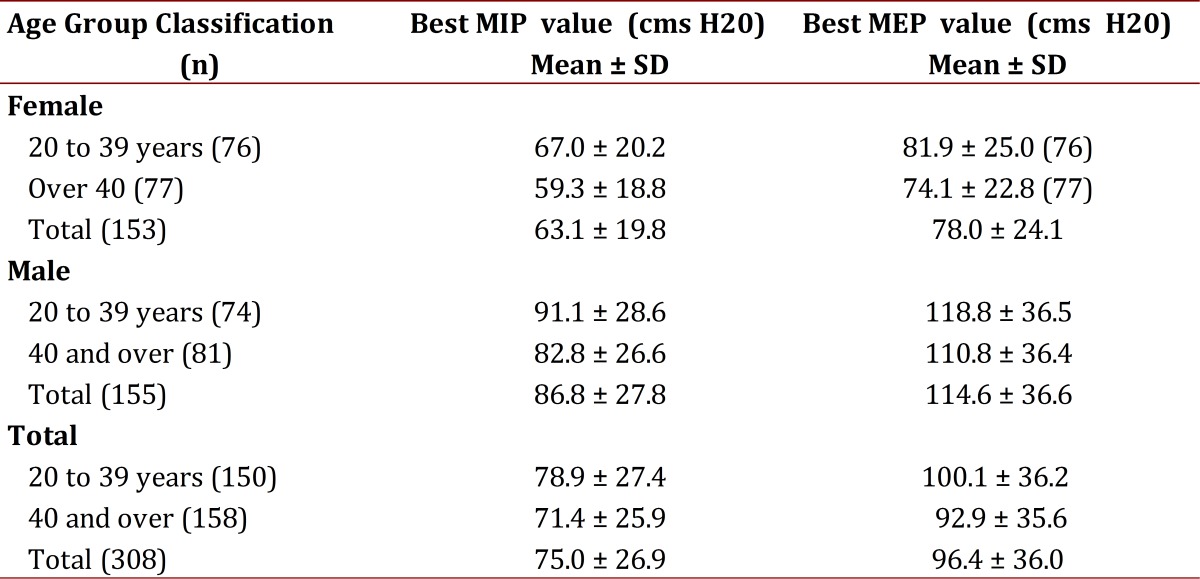
Maximal inspiratory and expiratory pressure according to age group and gender Manizales Colombia 2011

These factors affect biological aspects, in this case respiratory muscle strength, which is the result of a gradual adaptation to the environment in which one lives^8^ ^,^ ^15^ ^,^ ^16^ at least two classification parameters produced statistically significant differences in MEP values (F= 7.46, *p*= 0.001). At an alpha error of 5% there were no statistically significant differences (*p* =0.052) between the classification parameters with higher MEP values (mild obesity and overweight= 106.4 cms 105.7 cmsH20), however, these two have higher values compared with the normal BMI parameter value (90 cmsH20).

**Table 3:**
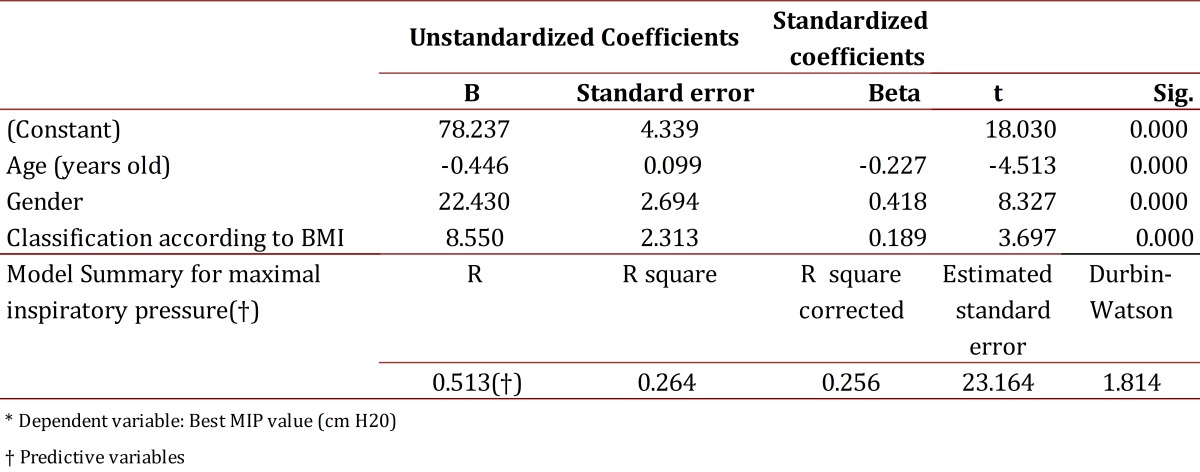
Coefficients and model summary for maximal inspiratory pressure ± Manizales Colombia

A multiple linear regression model was estimated in order to predict MIP and MEP values based on variables such as age, gender The highest coefficients of determination (r^2^) were taken for both MIP to MEP values; and the best model was tested for normality and homoscedasticity of waste, using the Kolmogorov-Smirnov and White tests respectively, it was also verified that the errors were normally distributed (K-S), equal to 0 and to be independent (Durbin Watson near 2). Finally, a multiple regression model was proposed to predict the dependence of the values obtained for maximal respiratory pressures respect to the independent variables with better r^2^ by a linear combination of the parameters used and the theoretical and practical formulation of the model obtained for values of maximum inspiratory and expiratory pressure was exposed.

The model used was as follows MIP: Overall test of the model:

MIP = *β1-β2 Age+β3Genre+β4BMIClassification+νi*

The model was statistically significant (F= 36,278, *p*= 0.000) MEP: Overall test of the model:

MEP =*β1+β2Genre +β3Weight+β4Height+ νi.*

The model was statistically significant (F= 50.16, *p*= 0.000)

**Table 4:**
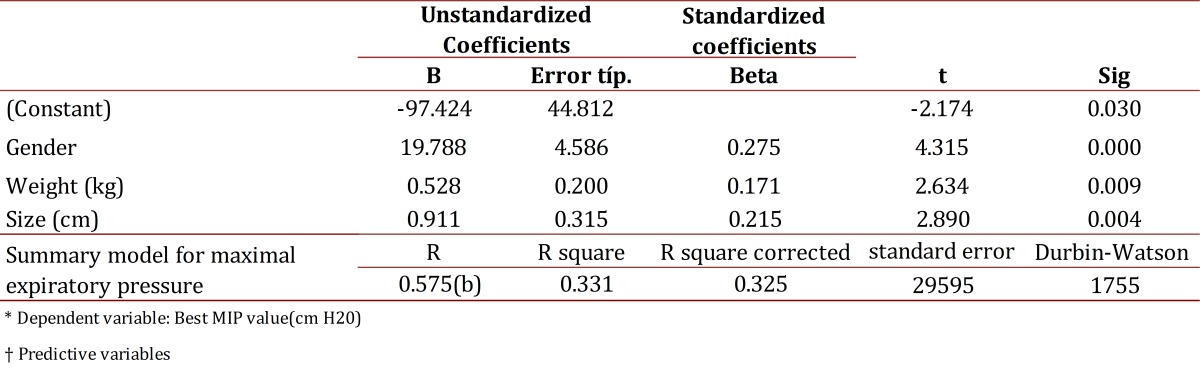
Coeficients and model summary for maximal expiratory pressure. Manizales Colombia 2011

## Discussion

Respiratory pressures combine the strength of the muscles of the chest and the contraction or relaxation of the chest wall. This means it is important to assess them using spirometric tests since there are dysfunctions that affect the respiratory muscles but not the bronchus itself. The results of this study show lower respiratory pressure values than those found in international studies, presenting averages of 75 cmH20 for MIP and 96.4 cmH20 for MEP when comparing average values and ranges of the whole sample. In contrast, other studies such as Black and Hyatt^5^exhibited average values of 94.5 and 175.5 cmH20 for MIP and MEP respectively, while Rodríguez *et al*.^14^, found average values of 90 and 127 cmH20 for MIP and MEP,. On the other hand, Simoes^10^ reported average values of 91 cmH20 for MIP and 98 cmH2O for MEP, Neder *et al*.^3^, found average values of 100 and 106 cm H20 for MIP and MEP, Parreira *et al*. ^2^, reported average values of 86 and 111 cmH20 for MIP and MEP, while Costa1 reported averages values of 82 and 102 cmH20 for MIP and MEP respectively.

The maneuvers for maximal respiratory pressures were performed on a voluntary basis, therefore the patient's mood, cooperation and understanding of how to carry out the tests could have influenced results. Another important aspect to consider is that all of the research studies mentioned, including this one, have not only used different measuring equipment but have also calibrated them under different conditions.^15^ ^,^ ^16^

**Figure 1:**
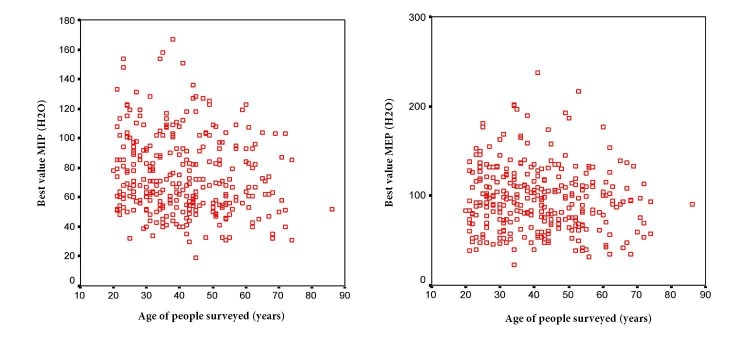
Ratio maximal inspiratory and expiratory pressure according to age Manizales

Women have lower MIP and MEP values when compared to men. Neder *et al*., reported MIP and MEP values of 115.3 and 125.23 cmH20 in men respectively, whereas in women values were 86.2 and 88 cmH20 respectively^3^. This could be due to anatomical, structural and hormonal differences.

Results show that MIP and MEP values decrease with age in both males and females. This data coincides with results found by McConnell *et al*.^9^ and Enright *et al*. ^18^, who stated that respiratory muscle strength decreases about 8-10% per decade after the age of 40. In contrast, Costa *et al*.^1^, found a negative correlation of MIP and MEP values with age in both men and women.

Results show that the greater the weight and the higher the body mass index (BMI) classification (mild obesity), the higher the respiratory pressure values. The influence of these variables may be due to the corresponding increase of muscle mass in relation to body weight, which is about 42% in men and 36% in women^19^

There is better androgenic hormone function and improved muscle protein synthesis when there is a weight gain accompanied by adequate nutrition. This makes muscle performance better in men than in women^20^. A BMI above 35 (moderate and severe obesity) does not mean greater muscle strength probably because these individuals present pulmonary restriction and mechanical disadvantage^21^. Height significantly influences MIP and MEP values. During infancy, bone growth is accompanied by an increase in muscle length in which multiplication of sarcomeres takes place and this in turn can generate more muscle strength in this respiratory case^22^. The variables in Costa1 study showed a positive correlation with weight and height in men, but with height only in women.

Wilson *et al* ^8^ and Harik-Khan's^23^ studies demonstrated that height was a negative predictor only in women and one of their studies ^23^ showed that weight was a positive predictor for both men and women.

Correlations were made between the variables of gender, age, weight, height, BMI and BMI Classification and maximum respiratory pressures in order to obtain prediction formulas. To determine the reference values for MIP the variables of age, gender and BMI classification were used while gender, weight and height were used to determine reference values for MEP. This regression formula differs from that proposed by Black and Hyatt^5^in which the best correlation was found when they used the age variable. In contrast, in Rodríguez's^14^ study carried out with a Venezuelan population, the variables included in the regression model were age and height.

Bruschi *et al*. ^24^, established equations for the Italian population, considering age and gender as well as body surface area as significant variables of their prediction equations. Costa *et al*.^1^ like Neder *et al*. ^3^, observed that age and gender had great predictive power and therefore proposed these variables for their new equations to determine respiratory muscle strength in the Brazilian population. The reference values of maximal respiratory pressures that were obtained by the proposed regression formulas were different from those identified in 5 studies by Black and Hyatt^5^, Rodríguez^14^, Bruschi *et al*. ^24^, Costa *et al*.^1^ and Neder *et al*. ^3^, that used prediction equations. This may be due to the fact that in each study a different device was used to measure results and also to the anthropometric differences of biotype, nutrition and physical activity among the studied populations.

The values found in this study were obtained from a reference population from Manizales, a Colombian city located at 2150 meters above sea level. Unlike in other research projects^25^, a pressure gauge and mouthpiece were used. This study strengthens the comprehensive explanatory network in relation to human body movement from cardiorespiratory and neuromuscular systems.

This has implications for both individual and collective interventions in healthy subjects. This can be done based on strategies promoting aerobic capacity and endurance as in any health condition with deficiencies in body functions or structures that affect the overall strength of the respiratory muscles from the therapeutic and pulmonary rehabilitation areas.

The standardization of the technique, the method and the procedure used to assess maximal respiratory pressures favors a universal practice. Similar studies in other Colombian regions are necessary to be able to generalize results for mean maximal respiratory pressures among the Colombian population.

## Conclusion

We present a first study that includes a group of predictive equations for maximal respiratory pressures from a sample population from the city of Manizales, Colombia. The results show lower maximal respiratory pressure values than those found in international studies. Gender and anthropometric characteristics (weight, height and BMI classification) are the variables that best explain MIP and MEP average values according to the proposed predictive models.

The established predictive values allow professionals to have standardized measures for decision-making that could be used as reference values to treat individuals with any health condition or disability.

### Recommendations of study

Similar studies in different Colombian regions are recommended to develop a predictive model for MIP and MEP from a multicenter study. This research demonstrated average MIP and MEP values in 2 main age groups (20-39, 40 and over). It is also recommended to extend the age range by 5-10 year periods which would allow the identification of differences between these groups and would also organize the sample in subgroups by BMI classification.
